# Diagnostic Accuracy of Blood-Based cfDNA/ctDNA Tests for Colorectal Cancer—A Systematic Review and Meta-Analysis

**DOI:** 10.3390/cancers18111752

**Published:** 2026-05-27

**Authors:** Jerry Henry Rose, Shivani Mattikalli, Aarav Bansal, Ruchita Ruturaj Jadhav, Yilin Song, George Kunnackal John, Ekta Gupta

**Affiliations:** 1 Department of Internal Medicine, University of Maryland Medical System, Baltimore, MD 21201, USA; smattikalli@som.umaryland.edu (S.M.); yilin.song@umm.edu (Y.S.); 2 Gilman School, Baltimore, MD 21210, USA; abansal31@students.gilman.edu; 3 Department of Internal Medicine, Sinai Hospital of Baltimore, Baltimore, MD 21215, USA; rjadhav@lifebridgehealth.org; 4 Division of Gastroenterology, Department of Medicine, University of Maryland Medical System, Baltimore, MD 21201, USA; gjohn@som.umaryland.edu (G.K.J.); egupta@som.umaryland.edu (E.G.)

**Keywords:** cell-free DNA, circulating tumor DNA, colorectal cancer screening, liquid biopsy, diagnostic accuracy, meta-analysis, non-invasive cancer detection

## Abstract

**Background/Objective**: Blood-based assays for colorectal cancer (CRC) screening have emerged as promising non-invasive screening modalities, yet their real-world diagnostic performance across platforms remains unclear. Existing studies evaluating circulating tumor DNA (ctDNA) and cell-free DNA (cfDNA) based assays as non-invasive alternatives to stool-based tests vary substantially in methodology, population, and study design. In our systematic review and meta-analysis, we quantify the diagnostic yield of contemporary cfDNA/ctDNA for CRC detection through pooled sensitivity and specificity. **Methods**: A systematic search of diagnostic accuracy studies published through December 2025 was conducted by two researchers in PubMed and the Cochrane Library. Studies reporting sensitivity and specificity were included. Pooled proportions with the 95% confidence interval (CI) were calculated using a hierarchical bivariate random-effects model. Subgroup analyses were conducted stratified by cancer stage and advanced adenoma. (PROSPERO CRD420261290686). **Results**: Fifty-eight studies involving 63,309 subjects were included. The pooled sensitivity and specificity for colorectal cancer detection were 82.9% (95% CI: 79.9–85.5%) and 90.7% (95% CI: 89.2–92.0%), respectively. HSROC analysis demonstrated excellent overall diagnostic accuracy with an AUC of 0.937. Pooled sensitivity for advanced adenoma was suboptimal at 46% (CI: 32–59%). Detection sensitivity increased progressively by stage, from 70% (CI: 62–78%) in stage I CRC to 90% (CI: 87–92%) in stage IV CRC. **Conclusions**: These findings position next-generation cfDNA/ctDNA assays as promising, scalable, patient-friendly CRC screening tools with performance characteristics comparable to established non-invasive tests. Integration of cfDNA/ctDNA assays into population-level screening programs may substantially expand screening uptake by overcoming key barriers associated with stool-based modalities.

## Simple Summary

Colorectal cancer remains a leading cause of cancer-related death, yet many individuals do not complete recommended screening. Barriers to screening include the invasive nature of colonoscopy, as well as patient reluctance to complete stool-based tests due to discomfort, inconvenience, or aversion. Blood-based tests that detect tumor DNA in circulation offer a promising, less invasive alternative. In this study, we analyzed data from 58 studies to evaluate how well these blood-based tests detect colorectal cancer. We found that these tests perform well overall, particularly in identifying more advanced cancers, but are less sensitive for early-stage disease and precancerous lesions. These findings suggest that blood-based screening tests could improve screening uptake and complement existing methods, although further refinement is needed to enhance early detection.

## 1. Introduction

Colorectal cancer (CRC) remains the second leading cause of cancer-related mortality and morbidity worldwide, as the third most diagnosed malignancy [1]. Effective prevention and early detection are essential for reducing disease burden [2]. Despite the availability of multiple screening modalities, such as colonoscopies and non-invasive tests, approximately one-third of screening-eligible patients remain not up to date with CRC screening, contributing to avoidable late-stage diagnoses and excess mortality [2]. Colonoscopies remain the reference standard for CRC screening as they allow providers to directly visualize the colon and remove suspicious precancerous polyps [3]. Colonoscopies are associated with their own barriers, such as ensuring proper bowel preparation, transportation needs after sedation, fear and stigma of the procedure, and time off work [4]. Non-invasive alternatives, such as fecal immunochemical testing (FIT) and multitarget stool DNA (mtsDNA) assays, overcome some of the barriers of colonoscopy but also have their own challenges, such as aversion to handling stool [5] and low adherence to colonoscopy if positive [6]. Thus, additional accessible screening methods are needed to accurately screen for colorectal cancer across diverse populations.

Blood-based assays for CRC screening are a promising, rapidly expanded approach that may reduce barriers to screening and improve adherence [7]. Yet, their real-world diagnostic performance across platforms remains unclear. Blood-based screening tests have the potential to increase adherence rates compared to invasive screening methods because they are more acceptable to patients, more accessible, and easier to undergo [7]. Guardant Shield is currently the only FDA-approved blood-based screening test for CRC using cell-free DNA (cfDNA), supported by the ECLIPSE trial (NCT03108456) [8]. The Freenome test is a methylation-based circulating tumor DNA (ctDNA) test that is still pending FDA approval and was evaluated in the PREEMPT trial (NCT04369053) [9]. In addition to these, other countries have developed additional cfDNA and ctDNA tests and predictive models, which we have included in our study.

Cell-free DNA (cfDNA) consists of short extracellular nucleic acid fragments released into the blood during physiological processes such as immune response, coagulation, aging, and oncogenesis [7,10]. Circulating tumor DNA (ctDNA is a tumor-derived subset of cfDNA that carries specific molecular alterations, including methylation signatures, copy number alterations, and fragmentomics abnormalities [11]. In multiple cancer types, including CRC, the concentration of cfDNA is significantly higher in plasma samples from cancer patients than in those from healthy patients [12].

Modern cfDNA-based screening assays leverage multiomic frameworks that integrate complementary genetic mutations with epigenetic information—predominantly DNA methylation patterns, as well as fragmentomic features reflecting chromatin accessibility and structural genomic alterations, such as chromosomal rearrangement and copy number variation- to improve diagnostic accuracy [11]. Emerging plasma DNA assays use artificial intelligence-informed, cancer-specific signatures derived from next-generation sequencing-based targeted bisulfite sequencing of >100,000 methylation sites and fragment ratios [7,10]. Because cfDNA may comprise less than 1% of total circulating cfDNA, early detection historically remained challenging due to the low abundance of tumor-derived DNA relative to background hematopoietic and epithelial cfDNA [10,11,12]. However, advances in next-generation sequencing, methylation profiling, fragmentomics, and machine learning-based multi-omic analysis have substantially improved the ability to detect these low-frequency tumor-derived signals.

Prior meta-analyses have reported promising diagnostic performance for blood-based cfDNA or ctDNA assays, with pooled sensitivities ranging from 72–81% and pooled specificities of 91–93% for colorectal cancer detection [13,14]. They also identify limitations, including reduced sensitivity for early-stage disease and for advanced adenoma detection [14]. Furthermore, several large prospective validation studies, including ECLIPSE and PREEMPT, have recently been published. However, to our knowledge, prior meta-analyses did not comprehensively combine both cfDNA and ctDNA assays, nor did they perform pooled subgroup analyses stratified by individual cancer stage and advanced adenoma detection. Therefore, we performed a comprehensive systematic review and meta-analysis of observational studies to quantify the diagnostic yield of contemporary cfDNA and ctDNA assays for colorectal cancer detection through pooled sensitivity and specificity. By incorporating recently published large-scale validation studies, global patient populations, stage-specific pooling, and advanced adenoma subgroup analyses, this study provides the most comprehensive contemporary synthesis of blood-based cfDNA/ctDNA screening performance for colorectal cancer through December 2025.

## 2. Materials and Methods

### 2.1. Search Strategy and Study Selection

A comprehensive literature search across PubMed and the Cochrane Library was conducted from database inception through 1 December 2025. Search terms included combinations of the following keywords and MeSH headings: (‘cell-free DNA’ OR cfDNA OR ‘circulating tumor DNA’ OR ctDNA OR liquid biopsy) AND (‘colorectal cancer’ OR CRC OR colorectal neoplasm) AND (screening OR detection OR diagnosis). Reference lists of included studies and relevant reviews were also manually screened to identify additional eligible studies. No formal language restrictions were applied during database searching; however, all included studies were available in English-language full text. This meta-analysis was conducted in accordance with the Preferred Reporting Items for Systematic Reviews and Meta-Analyses (PRISMA) 2020 guidelines and was registered with PROSPERO (CRD420261290686). Publication bias was assessed using the funnel plot asymmetry test, appropriate for diagnostic test accuracy meta-analyses.

We included studies that met the following criteria: (1) prospective or retrospective cohort studies, cross-sectional studies, or case–control studies; (2) reporting the diagnostic accuracy of cfDNA/ctDNA; (3) involving patients undergoing average-risk diagnostic or referral screening; and (4) colonoscopy with histopathological confirmation as the reference standard for CRC and advanced neoplasia. We excluded studies that (1) evaluated non-DNA biomarker assays, even if blood-based, such as circulating proteins, proteomic panels, other protein-based biomarkers, microRNA, metabolites, or exosomes; (2) reported insufficient data; (3) focused on prognosis or recurrence surveillance rather than screening; (4) were preclinical studies; or (5) were non-original research, such as reviews, editorials, or consensus statements without original accuracy data.

Screening and selection were performed first by title and abstract, and then by full-text content. Two authors (JHR and SM) independently extracted baseline characteristics reported in Table S1 and outcomes using prespecified criteria for search, data extraction, and quality assessment. Disagreements were resolved by consensus among three authors (JHR, SM, and EG). All data were manually collected into a standardized data collection sheet. Collected information included study title, publication year, study design, country, patient demographics, number of patients and controls, and diagnostic performance measures (true positives, false positives, false negatives, true negatives).

Study quality was assessed using the Quality Assessment of Diagnostic Accuracy Studies-2 (QUADAS-2) tool. Four domains were evaluated: patient selection, index test, reference standard, and flow and timing. Two authors (JHR and SM) independently rated each domain as low risk, unclear risk, or high risk of bias, and divergence was discussed until an agreement was reached. Inter-reviewer agreement for QUADAS-2 was substantial (Cohen’s κ = 0.81), and disagreements were resolved through consensus discussion with a third reviewer (EG). Studies were not excluded based on quality assessment; however, risk of bias was considered in interpreting the results.

The primary outcome of the analysis was the diagnostic accuracy of blood-based cfDNA/ctDNA tests, as measured by sensitivity (the ability to correctly identify CRC-positive individuals) and specificity (the ability to correctly identify CRC-negative individuals). The secondary outcomes were sensitivity for advanced adenoma (AA) and stage-specific sensitivity (Stage I-IV). Advanced adenoma was defined as any tubular lesion >10 mm or any adenoma with high-grade dysplasia, villous features, or carcinoma in situ.

### 2.2. Statistical Analysis

Pooled diagnostic accuracy estimates were calculated using a hierarchical bivariate random-effects model (Reitsma method), which models sensitivity and specificity while accounting for their correlation and between-study heterogeneity. The hierarchical bivariate model accounts for potential threshold effects arising from differences in assay positivity cutoffs across studies. Forest plots of sensitivity, specificity, and diagnostic odds ratios were generated to present study-level estimates; heterogeneity (Q, τ^2^, I^2^) was assessed. Hierarchical summary receiver operating characteristic (HSROC) curves were constructed to evaluate overall diagnostic performance, and the area under the curve (AUC) was calculated. Continuity correction was applied when necessary for studies with zero-cell counts.

Between-study heterogeneity was assessed using Cochran’s Q test, the I2 statistic (interpreted as low, moderate, and high at approximately 25%, 50%, and 75%, respectively), and Tau2 to estimate between-study variance. Accordingly, pooled estimates were interpreted as summary measures across heterogeneous conditions rather than precise predictors of performance in any single clinical setting.

Subgroup analyses were conducted to further explore heterogeneity by stratifying studies by cancer stage and advanced adenoma status, thereby allowing for a more clinically meaningful understanding of assay performance across the spectrum of colorectal neoplasia. Twenty-two studies reporting outcomes for advanced adenoma were included in the analysis. For stage-specific analyses, 27 studies reporting individual stage I–IV were included. Studies reporting grouped stage sensitivities (I–II or III–IV) were not disaggregated and were therefore excluded from stage-specific pooling. When raw counts were available, individual-stage data were analyzed. A meta-analysis of both subgroups was feasible given the number of included studies.

Exploratory bivariate diagnostic meta-regression analyses were performed to investigate sources of between-study heterogeneity. Prespecified study-level moderators included study design, assay platform, geographic region, and QUADAS-2 high-risk status. Meta-regression analyses were conducted within a hierarchical bivariate framework using likelihood ratio testing to assess moderator significance.

Sensitivity analysis was performed by excluding studies classified as high risk of bias according to the QUADAS-2 assessment and by repeating the primary hierarchical bivariate meta-analysis to evaluate the robustness of the pooled diagnostic estimates.

Publication bias was evaluated using the funnel plot asymmetry test, which is appropriate for diagnostic test accuracy meta-analyses. All analyses were performed in RStudio (v2025.09.2+418) with the mada and meta packages.

## 3. Results

### 3.1. Study Selection and Characteristics

Fifty-eight studies contributed to the pooled sensitivity analysis and fifty-seven to the pooled specificity analysis.

**Figure 1:**
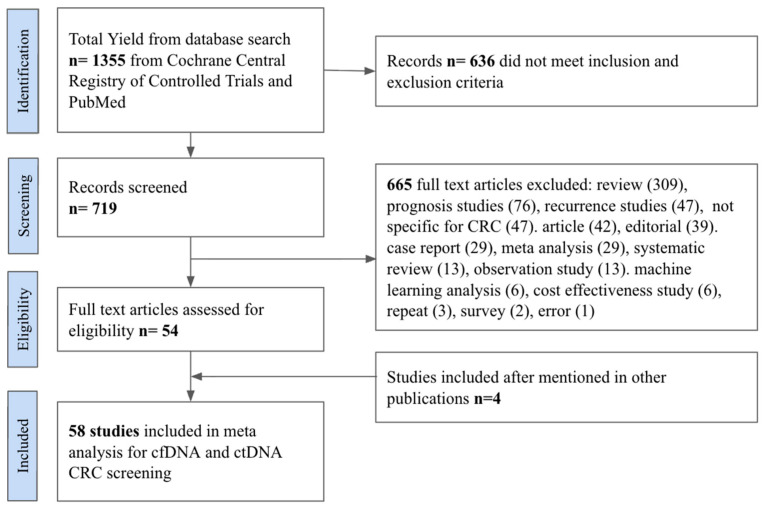
PRISMA 2020 flow diagram for cfDNA and ctDNA CRC screening summarizing eligible studies through 3 December 2025.

A total of 1355 studies were initially screened, 636 of which were excluded from the meta-analysis for not meeting the inclusion or exclusion criteria. Of the 719 studies screened, 665 were excluded for various reasons, including reviews, studies reporting on prognosis or cancer recurrence, studies not specific to colorectal cancer, and other reasons listed in the PRISMA diagram. 4 studies identified through reference screening were included, bringing the total to 58 studies in our meta-analysis (Figure 1). The studies were conducted across 14 different countries. The majority of the studies were conducted in China (*n* = 29) followed by the United States (*n* = 13). More than half of the studies were cohort validation studies of a liquid biopsy model (62.1%). The remaining were case–control studies (37.9%). Study characteristics are presented in Supplementary Table S1. All studies involved quantitative analysis of cfDNA or ctDNA [8,9,15,16,17,18,19,20,21,22,23,24,25,26,27,28,29,30,31,32,33,34,35,36,37,38,39,40,41,42,43,44,45,46,47,48,49,50,51,52,53,54,55,56,57,58,59,60,61,62,63,64,65,66,67,68,69]. 

### 3.2. CRC Detection Sensitivity

**Figure 2:**
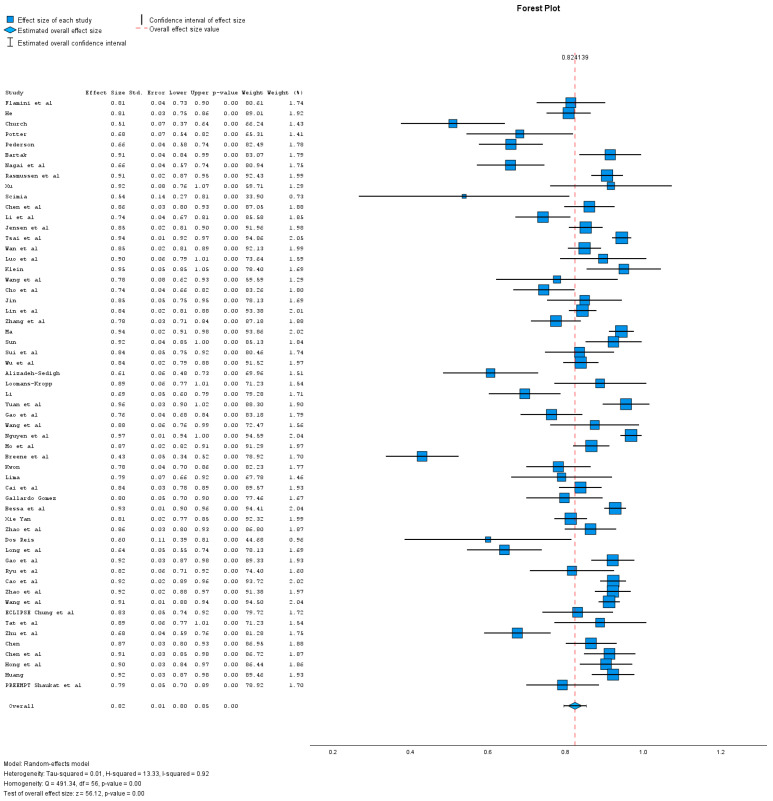
Forest Plot showing Sensitivity across included studies of cfDNA/ctDNA for colorectal cancer detection. Pooled estimates were calculated using a random-effects model. Horizontal lines represent 95% confidence intervals, and the size of each square reflects study weight. Substantial heterogeneity was observed across all studies [7,8,15,16,17,18,19,20,21,22,23,24,25,26,27,28,29,30,31,32,33,34,35,36,37,38,39,40,41,42,43,44,45,46,47,48,49,50,51,52,53,54,55,56,57,58,59,60,61,62,63,64,65,66,67,68,69].

Study-level sensitivity estimates are shown in Figure 2. Using the hierarchical bivariate random-effects model, pooled sensitivity for CRC detection was 82.9% (95% CI: 79.9–85.5%). Heterogeneity was very high (I^2^ = 92%).

### 3.3. Specificity for Non-Neoplastic Findings and/or Normal Colonoscopy

**Figure 3:**
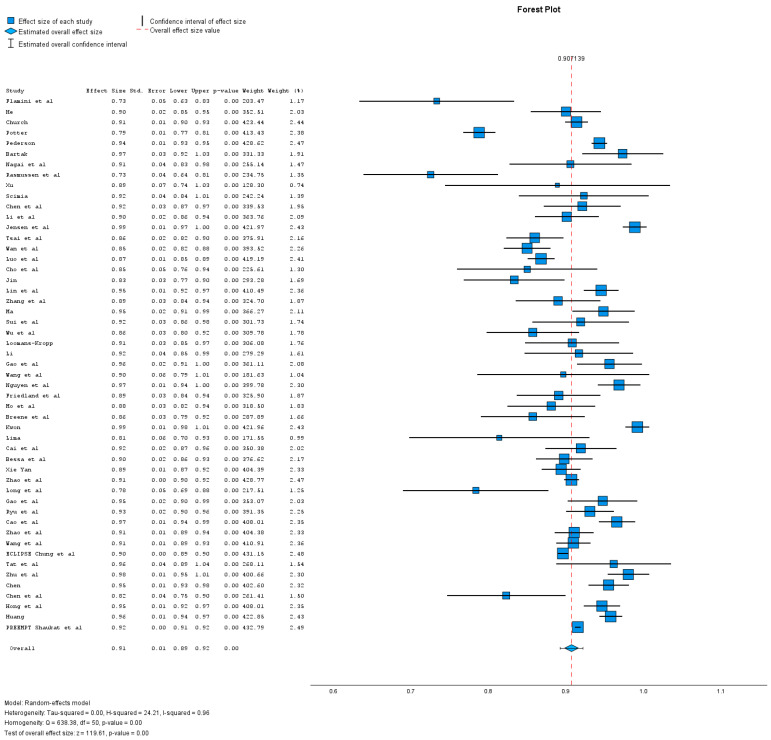
Forest plot showing specificity across included studies of cfDNA/ctDNA for non-neoplastic findings and/or normal colonoscopy. Pooled estimates were calculated using a random-effects model. Horizontal lines represent 95% confidence intervals, and the size of each square reflects study weight. Substantial heterogeneity was observed across all studies [7,8,15,16,17,18,19,20,21,22,23,24,25,26,27,28,29,30,31,32,33,34,35,36,37,38,39,40,41,42,43,44,45,46,47,48,49,50,51,52,53,54,55,56,57,58,59,60,61,62,63,64,65,66,67,68,69].

Pooled specificity was 90.7% (95% CI: 89–92%), indicating a low false-positive rate across the included studies and a strong ability to correctly identify individuals without colorectal cancer (Figure 3). Substantial between-study heterogeneity (I^2^ = 96%) was observed despite narrow pooled confidence intervals.

### 3.4. HSROC Analysis

**Figure 4:**
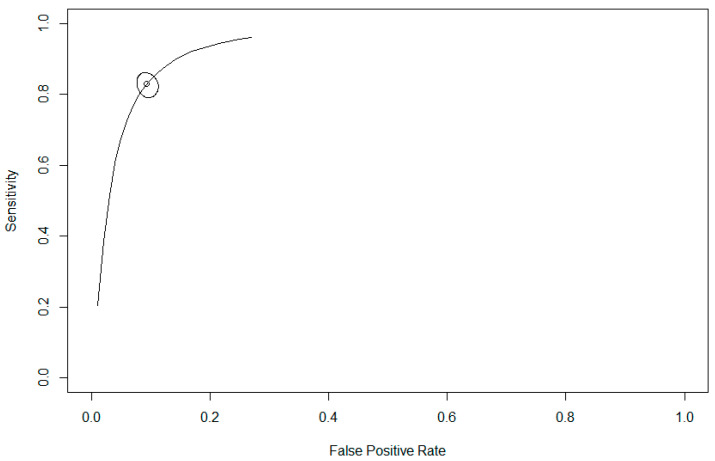
Hierarchical summary receiver operating characteristic (HSROC) curve for blood-based cfDNA/ctDNA assays in colorectal cancer detection. Each point represents an individual study estimate plotted by sensitivity and false-positive rate. The curve summarizes overall diagnostic performance using a hierarchical bivariate random-effects model. The area under the curve (AUC) was 0.938, indicating excellent overall discriminatory ability.

HSROC analysis demonstrated excellent overall diagnostic performance, with an AUC of 0.937 (Figure 4). Study estimates cluster predominantly in the high-sensitivity, low-false-positive-rate region, supporting a robust overall discriminative ability across platforms.

### 3.5. Subgroup Analysis: Advanced Adenoma Detection Sensitivity

**Figure 5:**
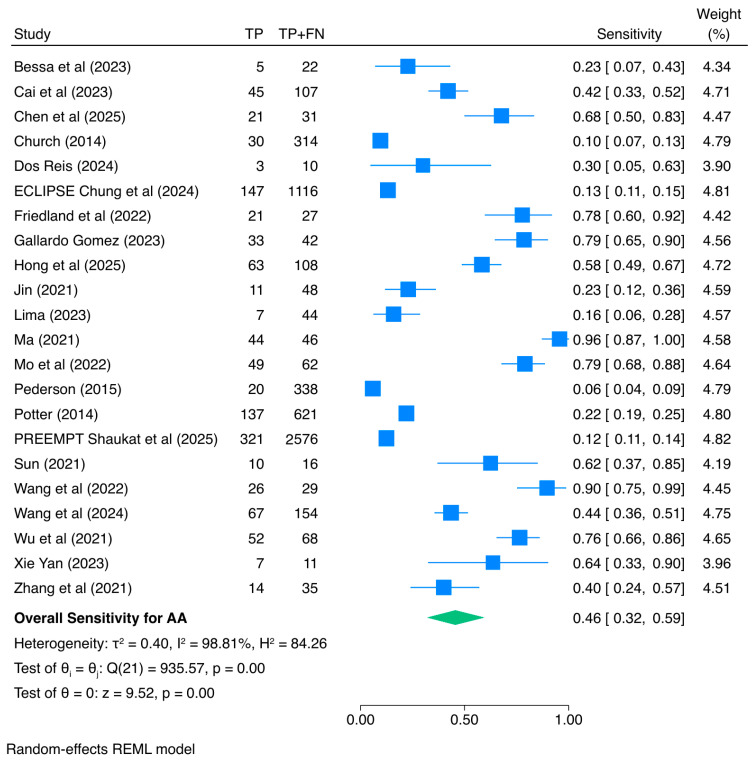
Forest plot showing the pooled sensitivity of cfDNA/ctDNA assays for detecting advanced adenomas. Estimates were derived using a random-effects model. Considerable heterogeneity was observed, reflecting variability in assay platforms, study populations, and diagnostic thresholds [7,8,17,18,19,24,34,36,37,38,40,46,48,49,53,55,56,58,63,64,67,68].

22 studies included data on the ability to detect advanced adenoma. Pooled sensitivity was 46% (95% CI: 32–59%) (Figure 5). Between-study heterogeneity was very high (I^2^ = 99%), similar to that observed in CRC.

### 3.6. Subgroup Analysis: Stage-Specific Sensitivity

**Figure 6:**
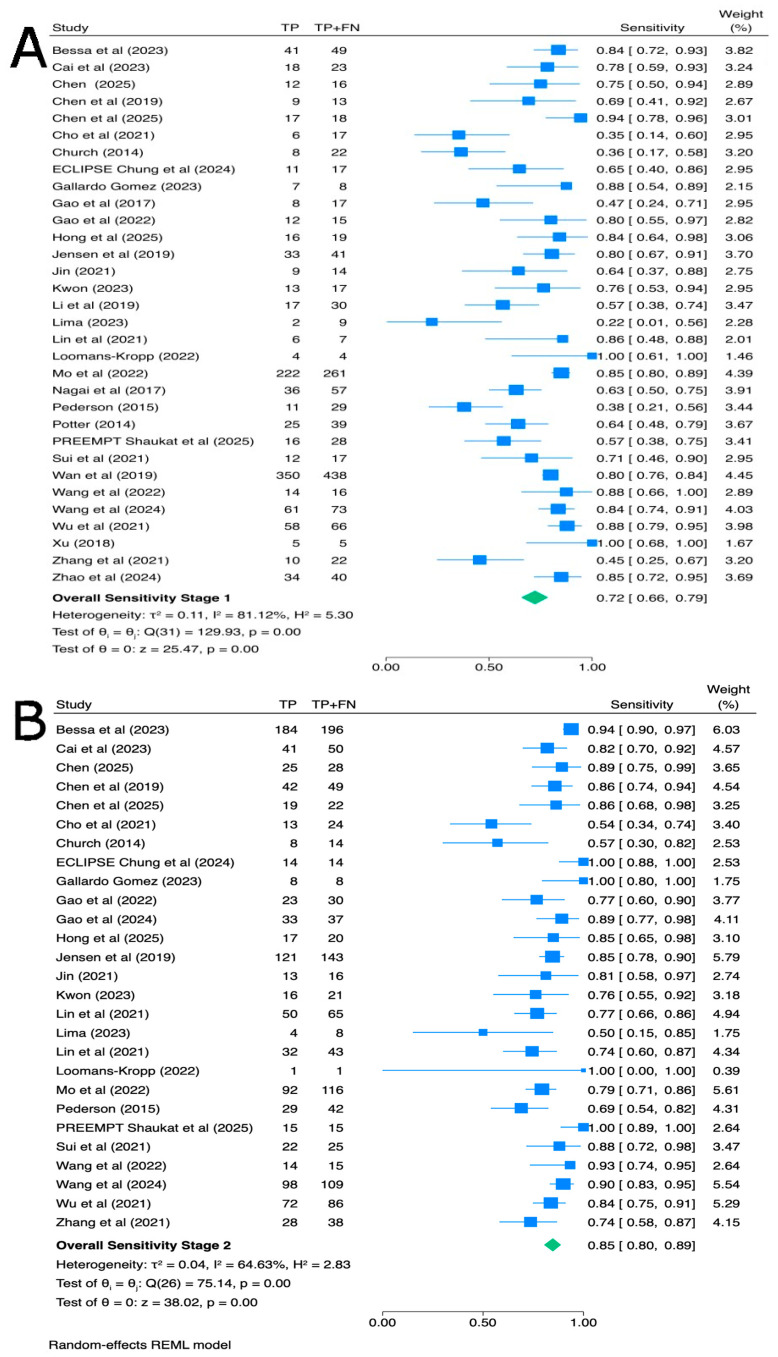

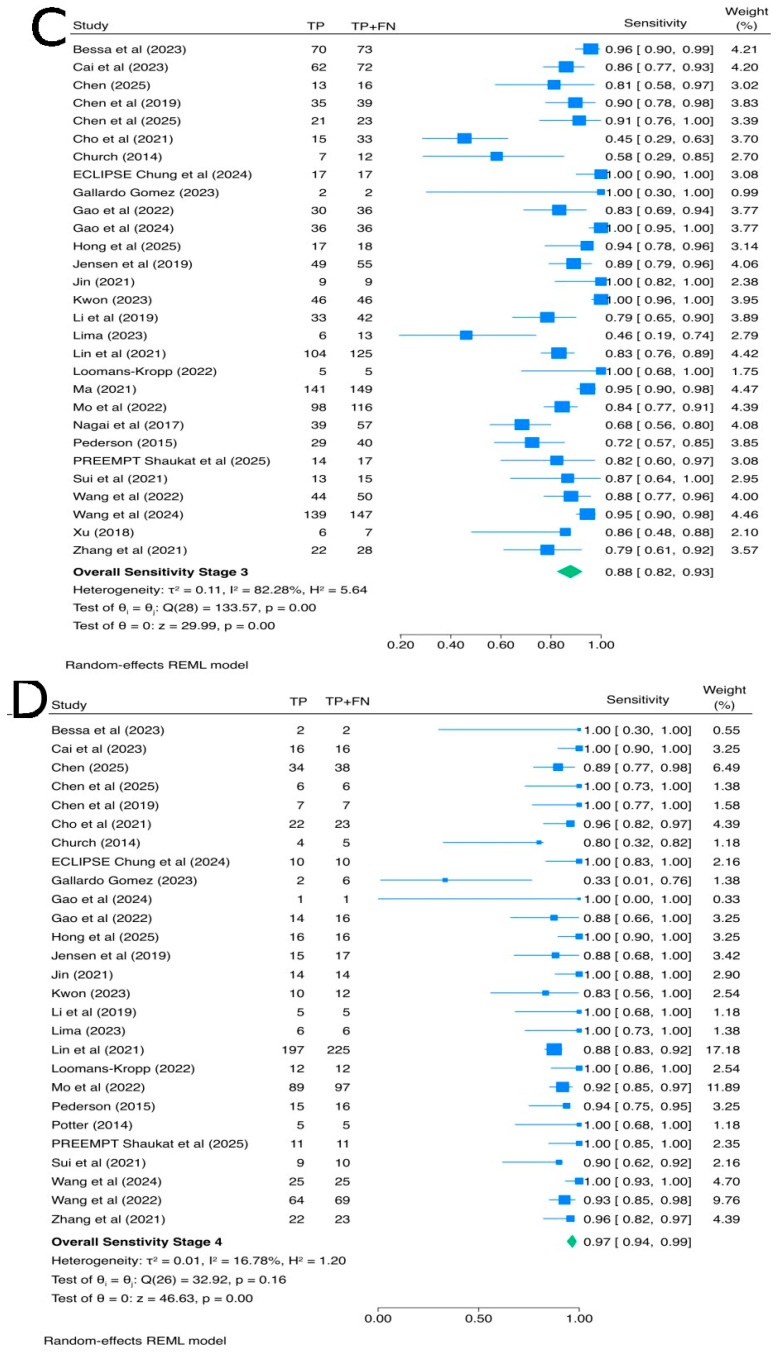
Forest plots demonstrating stage-specific sensitivity of cfDNA/ctDNA assays for CRC. (**A**) Stage I, (**B**) Stage II, (**C**) Stage III, and (**D**) Stage IV. Sensitivity increased progressively with advancing cancer stage, consistent with increased tumor DNA shedding in later-stage disease. Pooled estimates were calculated using a random-effects model [7,8,17,18,19,25,27,33,34,35,36,39,40,42,45,46,49,51,53,54,55,57,60,63,66,67,68].

To understand the role of cfDNA concentration in CRC progression, we stratified the CRC cohorts by cancer stage. 27 of the 58 studies reported data on individual cancer stage detection rates. Impressively, the detection rate in stage I CRC reached 70% (95% CI: 62–78%), increasing to 82% sensitivity (95% CI: 77–86%) in stage II CRC, to 85% (95% CI: 79–89%) in stage III CRC, and to 90% (95% CI: 87–92%) in stage IV CRC (Figure 6).

### 3.7. Diagnostic Odds Ratio, Positive Likelihood Ratio, Negative Likelihood Ratio

**Figure 7:**
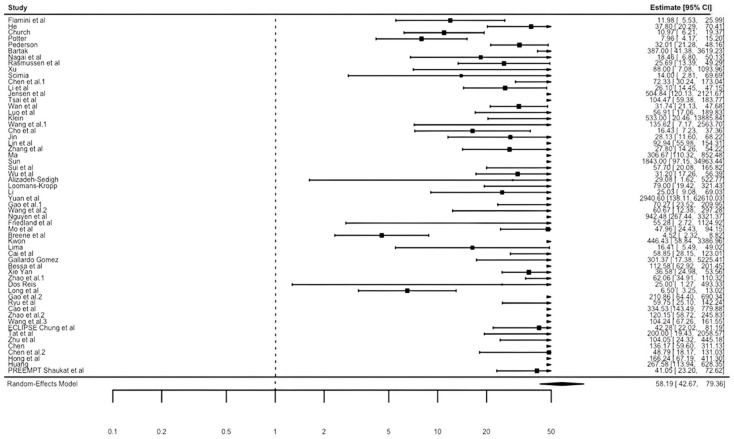
Forest plot of diagnostic odds ratios for cfDNA/ctDNA assays across included studies. The pooled DOR demonstrates the test’s strong overall discriminatory ability. Random-effects modeling was used to account for between-study heterogeneity [7,8,15,16,17,18,19,20,21,22,23,24,25,26,27,28,29,30,31,32,33,34,35,36,37,38,39,40,41,42,43,44,45,46,47,48,49,50,51,52,53,54,55,56,57,58,59,60,61,62,63,64,65,66,67,68,69].

The pooled diagnostic odds ratio is very high at 58.19, indicating substantially higher odds of testing positive among affected individuals than among controls (Figure 7). Using pooled sensitivity and specificity, we derived the positive and negative likelihood ratios. The derived positive likelihood ratio is 8.8, and the derived negative likelihood ratio is 0.19.

### 3.8. Quality Assessment

**Figure 8:**
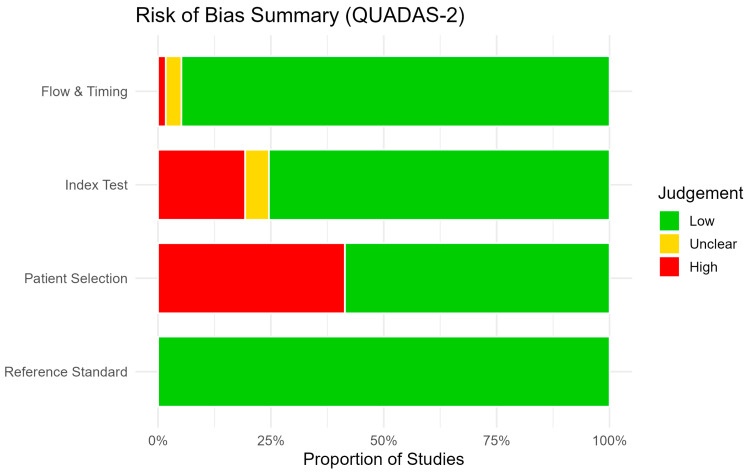
Risk of bias summary of included studies. QUADAS-2, Quality Assessment of Diagnostic Accuracy Studies-2.

Study quality was assessed using the QUADAS-2 tool, and the results are summarized (Figure 8). Overall, most studies demonstrated a low risk of bias across domains, indicating generally acceptable methodological quality. Concerns were prominent in the patient selection domain, with a substantial proportion of studies employing case–control or enriched-cohort designs (*n* = 24). In the index test domain, 11 studies derived diagnostic thresholds during assay development rather than applying predefined thresholds. All included utilized colonoscopy with histological confirmation.

### 3.9. Meta-Regression

Given the substantial heterogeneity observed across studies and the variability identified in QUADAS-2 assessment, exploratory bivariate diagnostic meta-regression was performed to evaluate sources of between-study heterogeneity. Assay platform was identified as a significant contributor to heterogeneity (χ^2^ = 28.51, df = 8, *p* < 0.001). In contrast, study design (case–control versus cohort), geographic region, and QUADAS-2 high-risk status were not statistically significant moderators of heterogeneity (all *p* > 0.05) (Table 1).

### 3.10. Leave-Out Sensitivity Analysis

Sensitivity analysis was conducted to evaluate the robustness of the pooled effect estimates. Excluding studies classified as high risk of bias on the QUADAS-2 (*n* = 29) demonstrated a pooled diagnostic performance similar to that of the primary analysis. Among low-risk studies, pooled sensitivity was 83.8% (95% CI: 79.7–87.3%) and specificity was 90.8% (95% CI: 88.9–92.4%), with an AUC of 0.941 (Table 2). These findings suggest that the primary pooled estimates were robust and studies at high risk of bias did not substantially alter the results.

### 3.11. Publication Bias

**Figure 9:**
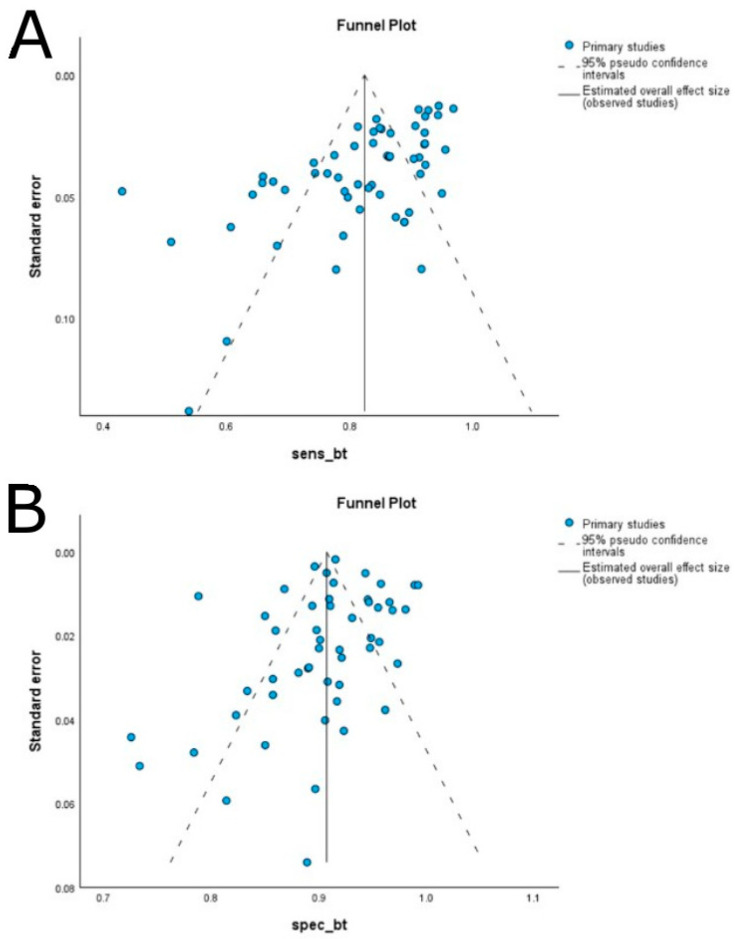
Funnel Plot for Publication Bias Assessment of (**A**) Sensitivity and (**B**) Specificity. Each point represents an individual study plotted by effect size (Hedges’ g) against standard error. The vertical line represents the overall estimated effect (ϑ_iv_), and the pseudo 95% confidence interval bounds are shown as dotted lines. Visible asymmetry was observed, with clustering of studies on the right and a notable spread on the left. An Egger-type regression test demonstrated significant funnel plot asymmetry (z = 3.48, *p* = 0.00005), suggesting possible publication bias or small-study effects.

Funnel plot asymmetry using an Egger-type regression test (Figure 9) demonstrated significant asymmetry (z = 3.48, *p* = 0.00005). However, asymmetry in diagnostic accuracy meta-analyses may also reflect heterogeneity across assay platforms, diagnostic thresholds, study populations, and disease-stage distributions. Publication bias may modestly overestimate diagnostic accuracy estimates, but should be interpreted in the context of substantial methodological heterogeneity across studies.

Across all analyses, effect precision was high (SE < 0.02), and random-effects models converged robustly. Forest plots visually demonstrated tight clustering of study-level estimates around pooled effects.

## 4. Discussion

This systematic review and meta-analysis provide the most comprehensive synthesis of the literature on blood-based screening modalities for colorectal cancer. Based on our analysis of all available data on the subject, we herein provide evidence supporting the potential clinical utility of the assay as a practical approach for CRC diagnosis. This meta-analysis demonstrates that blood-based cfDNA/ctDNA testing achieves an overall sensitivity of 82.9% and specificity of 91% for CRC, although the findings were accompanied by high between-study heterogeneity. Subgroup analysis demonstrated an increasing detection rate with advancing tumor stage. However, its sensitivity for advanced adenoma is only 46%. Despite these significant findings, heterogeneity remained substantial within the stage I-III and advanced adenoma subgroups, indicating variability in study sizes and effect magnitudes across studies. In contrast, the stage IV subgroup exhibited comparatively lower heterogeneity, suggesting greater consistency, which aligns with recent reviews of novel cancer screening tools [7,8,12].

This substantial variability is attributable to differences in assays and study designs, biomarkers, and analytic thresholds across cfDNA/ctDNA platforms. These sources of heterogeneity are intrinsic to rapidly evolving liquid biopsy technologies and underscore the need for pooled estimates rather than single-study performance metrics.

The robustness of the pooled estimate was evaluated through sensitivity analysis. An analysis restricted to low-risk studies yielded nearly identical pooled estimates with AUC changing the AUC from 0.937 to 0.941, supporting the robustness of the primary findings and suggesting that methodological bias did not substantially influence overall diagnostic performance.

Compared with previous systematic review and meta-analysis reporting on blood-based assays for CRC, our analysis demonstrated high precision in effect size estimates. The prior meta-analysis by Kumar et al. (2025) [13] reported data on cfDNA from 16 studies involving 15,591 patients. We included 58 studies covering 63,309 patients. Due to the larger number of studies, narrower confidence intervals and greater heterogeneity were observed, in contrast with those from previous studies.

Over the years, cfDNA/ctDNA technology has advanced significantly. With sensitivity rising from 65% to now 90% and consistently high specificity, diagnostic accuracy now rivals commonly accepted standards of practice. The pooled sensitivity for cfDNA/ctDNA is 82%, compared with 67.3% for FIT and 93.9% for mtsDNA [70,71]. The pooled specificity for these blood-based assays is 91%, compared with 90.6% for FIT and 94.8% for mts-DNA [71]. For CRC detection, cfDNA/ctDNA outperforms FIT but falls meaningfully short of mtsDNA testing. Notably, the sensitivity for advanced precancerous lesions remains limited across all non-invasive modalities, 46% for cfDNA/ctDNA in the present meta-analysis, 43.4% for next-generation mtsDNA, and only 23.3% for FIT [71].

The limited detection of advanced adenomas likely reflects a fundamental biological constraint rather than a deficiency unique to blood-based testing. Precancerous and early-stage lesions shed substantially less tumor DNA into circulation than invasive cancers because they are smaller, less vascularized, and undergo less cellular turnover. As a result, ctDNA fractional abundance in patients with advanced adenoma is often extremely low and may be indistinguishable from cfDNA released during normal hematopoietic and epithelial cell turnover, placing the tumor-derived signal near the analytical detection limits of current assays [72]. This biological limitation explains why sensitivity improves with advancing tumor stage, as larger, more invasive tumors release greater amounts of detectable DNA into the bloodstream. Although ongoing advances in fragmentomics, methylation profiling, and sequencing depth may improve early lesion detection, reduced sensitivity for premalignant lesions remains an important limitation of blood-based screening methods.

Blood-based cfDNA/ctDNA offers several advantages over other non-invasive colon cancer screening methods. Patient compliance is higher with blood-based samples because patients prefer them to stool-based tests [12]. These tests can be used to supplement measures to address the health equity gap among those who are not up to date with colonoscopy screening. cfDNA/ctDNA testing may offer its greatest real-world value in addressing the persistent problem of non-adherence to CRC screening. CRC mortality remains high in the United States due to non-adherence to recommended tests, with barriers such as cost, transportation, and stigma contributing to this issue, and cfDNA blood-based testing offers a promising alternative for improving accessibility and compliance, especially for those unwilling or unable to undergo more invasive procedures. cfDNA/ctDNA can be used to supplement (1) patients with a negative FIT test, but have high risk factors (family history), (2) those with a positive FIT test, but refuse colonoscopy, and (3) those who refuse to provide a stool test for FIT. When applied at the population level, this can optimize selection and reduce the number of colonoscopies needed to detect a single CRC. Accordingly, blood-based cfDNA/ctDNA testing is best positioned not as a universal first-line screening tool, but as a targeted intervention for the estimated one-third of eligible individuals who remain unscreened due to barriers to colonoscopy or stool-based testing.

Subgroup analysis by cancer stage provided insight into the assay’s clinical utility. We observed a positive association between sensitivity (detection rate) and cancer stage. The ability to detect later-stage adenocarcinomas is higher than for early-stage adenocarcinomas. It may be that precancerous lesions and early-stage tumors shed small amounts of DNA into the bloodstream that fall below the detection thresholds of standard blood collection volumes. Senescent tumor cells in cancer patients may undergo necrosis or enhanced apoptosis, with cell fragments in the plasma [67].

The pooled diagnostic odds ratio (DOR) of 58.19 observed in this analysis indicates a strong overall discriminatory ability of cfDNA/ctDNA assays for colorectal cancer detection, substantially exceeding the conventional threshold of 10, which is considered indicative of satisfactory diagnostic performance. The derived positive likelihood ratio (PLR) of 8.8 indicates that individuals with colorectal cancer are nearly 9 times more likely to have a positive cfDNA/ctDNA test result than those without disease, supporting the strong rule-in capability of these assays. Conversely, the negative likelihood ratio (NLR) of 0.19 indicates that a negative test substantially lowers, but does not eliminate, the probability of colorectal cancer. This modest NLR likely reflects the limited sensitivity of blood-based assays for early-stage cancers and advanced adenomas, in which tumor DNA shedding into the circulation remains low. Compared with established noninvasive screening modalities such as FIT and mtsDNA testing, cfDNA/ctDNA assays appear to favor specificity and confirmatory value over maximal sensitivity. These findings suggest that blood-based cfDNA/ctDNA testing may be best positioned as part of a complementary screening strategy rather than as a standalone rule-out test.

Our QUADAS-2 quality assessment revealed that many of the included studies used case–control and enriched-cohort designs rather than enrolling average-risk individuals from general screening populations. These study designs often compare patients with established colorectal cancer against healthy controls or selected non-neoplastic cohorts, which may artificially increase diagnostic separation between groups and overestimate test performance relative to real-world screening settings. This introduces the potential for spectrum bias, as assay sensitivity and specificity derived from advanced or clinically apparent cancers may not reflect performance in asymptomatic average-risk individuals with earlier-stage or biologically indolent lesions. Additionally, QUADAS-2 revealed that many studies developed thresholds for cancer rather than using a prespecified standard. Variability across studies in assay platforms, biomarker compositions, sequencing methods, threshold effects, and case selection contributed to heterogeneity in pooled estimates.

Exploratory meta-regression identified assay platform as a significant contributor to heterogeneity. The effect of assay platform on heterogeneity likely reflects technological variability across included cfDNA/ctDNA assays, including differences in methylation panels, fragmentomics, mutation-based assays, and multiomic machine learning models. Conversely, neither study design nor geographic region significantly explained heterogeneity, suggesting that assay methodology itself may be a more important determinant of diagnostic variability than differences in population source or observational design.

Funnel plot asymmetry testing using an Egger-type regression approach demonstrated significant asymmetry (*p* = 0.0005), suggesting possible publication bias or small-study effects. However, asymmetry in diagnostic meta-analyses may also reflect heterogeneity related to assay platforms, diagnostic thresholds, and study populations. The presence of significant funnel plot asymmetry should be interpreted cautiously, as diagnostic accuracy meta-analyses are particularly susceptible to heterogeneity arising from threshold effects, assay variability, and disease stage distribution, which may contribute to apparent small-study effects independent of true publication bias. Accordingly, pooled diagnostic accuracy estimates may be modestly overestimated and should be interpreted in the context of substantial methodological and clinical heterogeneity across studies.

Our review has many strengths, including a systematic literature search with well-defined inclusion criteria, the inclusion of high-quality, recent studies (most published after 2019), and a rigorous evaluation of study quality. First, this study represents one of the most comprehensive syntheses to date of circulating cfDNA and ctDNA-based assays for colorectal cancer detection, incorporating 58 studies across diverse patient populations. By including both tests, such as Shield, and emerging technologies, this analysis provides a comprehensive, globally encompassing assessment of blood-based screening methods and their performance. Additionally, this study goes beyond aggregate diagnostic accuracy by providing subgroup analysis, including stage-specific sensitivity and data on advanced adenomas. This analysis is particularly important, as early-stage detection and identification of precancerous lesions are key to CRC screening and, in turn, to reducing mortality rates. Finally, this study addresses a critical gap in the literature. As blood-based CRC screening tests move rapidly towards clinical implementation and federal regulatory approval, there is a need for a comprehensive analysis of their diagnostic performance, which this study provides.

An important factor in interpreting these findings is the evolving nature of cfDNA/ctDNA assays. Many studies have used machine learning-driven multiomic models and next-generation sequencing, in which diagnostic thresholds are derived during model training rather than fixed beforehand. This reflects the current stage of the field, where assay performance is still being optimized. Advances in fragmentomics, methylation profiling, and whole-genome sequencing have improved the detection of tumor-derived signals, especially in later stages. As a result, these assays are shifting from exploratory biomarkers to clinical diagnostic tools.

This meta-analysis has several limitations. The included studies varied in design and patient selection, encompassing cohort, cross-sectional, and case–control studies, which may introduce spectrum bias and lead to substantial statistical heterogeneity. Differences in biomarker composition and analytic thresholds also add to heterogeneity. Many studies were conducted in enriched or referral-based populations rather than true average-risk screening cohorts. As a result, pooled estimates of sensitivity and specificity may not reflect real-world performance in population-level CRC screening programs. Additionally, pooling multiple assay platforms may obscure platform-specific differences. Although funnel plot asymmetry testing suggested potential small-study effects or publication bias, formal correction methods such as trim-and-fill were not applied because these approaches are not well validated for diagnostic test accuracy meta-analyses and may be confounded by threshold-related heterogeneity. The incomplete reporting of 2 × 2 diagnostic tables in some studies limited the scope of secondary analyses. Finally, this analysis assessed diagnostic accuracy rather than downstream clinical effectiveness, and therefore does not directly address the impact on CRC incidence, mortality, or screening adherence.

## 5. Conclusions

In conclusion, this meta-analysis quantifies the diagnostic yield of blood-based cfDNA/ctDNA assays for colorectal cancer. Diagnostic accuracy for colorectal cancer was high, with the detection rate rising as tumor stage advanced. However, the sensitivity for advanced adenomas was suboptimal. Although it is a well-accepted non-invasive adjunct screening tool for patients, further optimization is still required to improve the detection of early-stage and precancerous lesions. The variability across studies reflects the evolving nature of this technology and underscores the need for assay standardization. As such, the test is currently suitable as a complementary option for individuals who are unwilling to undergo traditional screening, and cannot completely replace colonoscopy or stool-based screening.

## Figures and Tables

**Table 1:** Exploratory bivariate diagnostic meta-regression using hierarchical bivariate random-effects models.

Moderator	Likelihood Ratio Test (χ^2^)	df	*p*-Value
Study design (case–control vs. cohort)	0.354	2	0.838
Assay platform	28.510	8	<0.001
Geographic region	3.525	2	0.172
QUADAS-2 high-risk status	0.221	2	0.895

**Table 2:** Summary of Sensitivity Analysis. High-risk studies excluded: Flamini 2006 [15], Nagai 2017 [21], Rasmussen 2017 [22], Xu 2018 [23], Chen 2019 [25], Li 2019 [26], Klein 2021 [31], Wang 2021 [32], Cho 2020 [33], Lin 2021 [35], Sui 2021 [39], Wu 2021 [40], Alizadeh-Sedigh 2022 [41], Loomans-Kropp 2022 [42], Yuan 2022 [44], Gao 2022 [45], Nguyen 2022 [47], Kwon 2023 [51], Lima 2023 [52], Xie Yan 2023 [56], Zhao 2023 [57], Dos Ries 2024 [58], Gao 2024 [60], Ryu 2024 [61], Cao 2024 [62], Tat 2025 [64], Zhu 2025 [65], Chen 2025 [66].

Analysis	Sensitivity (95% CI)	Specificity (95% CI)	AUC
Primary analysis	82.9% (79.9–85.5)	90.7% (89.2–92.0)	0.937
Low-risk QUADAS-2 only	83.8% (79.7–87.3)	90.8% (88.9–92.4)	0.941

## Data Availability

The original contributions presented in this study are included in the article/Supplementary Material. Further inquiries can be directed to the corresponding author.

## Supplementary Materials

The following supporting information can be downloaded at: https://www.mdpi.com/article/10.3390/cancers18111752/s1, Table S1: Study Characteristics. Study-level data include design, assay type, and population characteristics. Diagnostic accuracy measures (TP, FN, FP, TN) were used to derive sensitivity, specificity, and likelihood ratios. Additional reported outcomes include stage-specific sensitivity and advanced adenoma detection. TP = true positives; FN = false negatives; FP = false positives; TN = true negatives [15,16,17,18,19,20,21,22,23,24,25,26,27,28,29,30,31,32,33,34,35,36,37,38,39,40,41,42,43,44,45,46,47,48,49,50,51,52,53,54,55,56,57,58,59,60,61,62,63,64,65,66,67,68,69].

## Abbreviations

The following abbreviations are used in this manuscript:

AA	Advanced Adenoma
CI	Confidence Interval
CRC	Colorectal Cancer
ctDNA	Circulating Tumor DNA
cfDNA	Cell-free DNA
DOR	Diagnostic Odds Ratio
FIT	Fecal Immunochemical Testing
MeSH	Medical Subject Headings
mt-sDNA	Multitarget stool DNA testing
PRISMA	Preferred Reporting Items for Systematic Reviews and Meta-analyses
REML	Random Effects Models
